# Outcomes of delayed versus early endoscopic intervention for acute biliary pancreatitis with non-severe acute cholangitis

**DOI:** 10.1186/s12893-022-01890-8

**Published:** 2022-12-26

**Authors:** Yunxiao Lyu, Shenjian Ye, Bin Wang

**Affiliations:** 1 Department of Hepatobiliary Surgery, Dongyang People’s Hospital, Affiliated Dongyang Hospital of Wenzhou Medical University, 60 West Wuning Road, Dongyang, 322100 Zhejiang People’s Republic of China; 2 School of Medicine, Shaoxing University, Shaoxing, 312000 Zhejiang People’s Republic of China

**Keywords:** Endoscopic retrograde cholangiopancreatography, Acute biliary pancreatitis, Acute cholangitis

## Abstract

**Background:**

Despite previous studies on endoscopic interventions in patients with acute biliary pancreatitis (ABP), the optimal time to perform endoscopic retrograde cholangiopancreatography (ERCP) for ABP with non-severe acute cholangitis (AC) remains controversial.

**Methods:**

We performed a retrospective cohort analysis of patients with concurrent ABP and non-severe AC. The patients were divided into two groups: those who underwent ERCP ≤ 72 h after admission (early ERCP group) and those who underwent ERCP > 72 h after admission (delayed ERCP group). The primary outcomes were the technical success rate and ERCP-related complications.

**Results:**

The study involved 164 patients (early ERCP, n = 70; delayed ERCP, n = 94) who were treated from 1 December 2 to 2016 to 12 December 2021. The patients’ baseline characteristics were not significantly different between the two groups. The technical success rate of ERCP was similar between the two groups (94.29% vs. 97.87%, *p* = 0.43). Morbidity was also similar between the two groups (*p* = 0.83). There was no significant difference in the total hospital stay (*p* = 0.13). However, the early ERCP group had a longer post-ERCP hospital stay (*p* < 0.001).

**Conclusion:**

This retrospective analysis showed that delayed ERCP performed > 72 h after admission has economic and safety outcomes similar to those of early ERCP for patients with concurrent ABP and non-severe AC.

## Introduction

Acute biliary pancreatitis (ABP) is thought to occur secondary to biliary obstruction, which is most frequently caused by bile duct stones [1]. ABP is one of the most prevalent digestive tract diseases in clinical practice, leading to a tremendous financial burden in human society. ABP often occurs in conjunction with acute cholangitis (AC). Endoscopists frequently perform endoscopic retrograde cholangiopancreatography (ERCP) for the treatment of ABP. Several studies have been conducted on this topic. Previous studies have demonstrated that early ERCP may be beneficial for ABP with severe cholangitis [2, 3]. However, the optimal time of performing ERCP for patients with ABP remains controversial [4, 5]. Several previous studies and guidelines have indicated that for patients with ABP, ERCP should be performed early (within 72 h) in patients without cholangitis and more urgently in patients with AC [5–7]. However, most cases of ABP are self-limiting, and up to 15% of stones may pass spontaneously during the early period of ABP [8]. Despite these previous studies and guidelines, the optimal time of performing ERCP for the treatment of ABP with non-severe AC remains controversial because of the ambiguous definitions of the timing of ERCP and cholangitis. We conducted a retrospective analysis to investigate the outcomes of early ERCP (≤ 72 h after admission) and delayed ERCP (> 72 h after admission) for treatment of ABP with non-severe AC.

## Materials and methods

### Patients

We retrospectively analyzed patients diagnosed as ABP with non-severe AC due to common bile duct stones from 1 to 2016 to 12 December 2021 using the electronic medical database of our hospital. The study was approved by the ethics committee of Dongyang People’s Hospital. AP was defined according to the American College of Gastroenterology guidelines: (1) abdominal pain consistent with the disease, (2) serum amylase and/or lipase greater than three times the upper limit of normal, and/or (3) characteristic findings from abdominal imaging, as well as abnormal liver enzymes[4]. Biliary pancreatitis was defined at least one of the following criteria: (1) gallstones or biliary sludge on imaging; (2) dilated CBD on imaging; (3) total bilirubin more than two times the upper limit of normal. The severity of ABP was determined according to the Bedside Index of Severity in Acute Pancreatitis (BISAP) score [9]. The definition and severity of acute cholangitis were determined according to the 2018 Tokyo Guidelines [10]. ERCP-related complications were assessed based on the American Society for Gastrointestinal Endoscopy guidelines [11]. All medical information was recorded in an electronic system. Data regarding age, sex, body mass index, comorbidities, American Society of Anesthesiologists (ASA) physical status, and laboratory findings were collected. Patients were excluded if they had not undergone ERCP or if they had undergone digestive tract reconstruction. Patients with obstruction without AC or AC due to stricture, tumor, stent were also excluded. The included patients were divided into two groups according the time at which ERCP was performed after admission: those who underwent ERCP ≤ 72 h after admission (early ERCP group) and those who underwent ERCP > 72 h after admission (delayed ERCP group). All patients received intravenous fluids and antibiotic treatment once acute cholangitis had been diagnosed.

### ERCP procedures

All ERCP procedures were performed by two expert endoscopists who had performed 200 ERCP per year with a standard therapeutic duodenoscope (Olympus JF-260; Olympus, Tokyo, Japan). All patients received topical pharyngeal anesthesia with 2% lidocaine followed by intravenous administration of midazolam for sedation and fentanyl for analgesia; the doses used were at the discretion of the endoscopist. Cannulation of the common bile duct was attempted with a conventional cannula with a guidewire. All patients were monitored continuously during the procedure by measurement of blood pressure, heart rate, respiratory rate, and arterial oxygen saturation.

### Outcome measures

The primary outcomes were the technical success rate and ERCP-related complications. The secondary outcomes were the hospital stay and patient cost. Technical success was defined as successful biliary cannulation and removal of stones from the bile duct. ERCP-related complications were defined according to the American Society for Gastrointestinal Endoscopy guidelines [11].

### Statistical analysis

Statistical analyses of the clinical data were performed using SPSS software version 26 (IBM Corp., Armonk, NY, USA). Nonparametric variables were analyzed with the Mann–Whitney U test, and categorical-type outcomes were analyzed with the chi-square test or Fisher’s exact test. A *p* value of < 0.05 was considered statistically significant. All authors had access to the study data and reviewed and approved the final article.

## Results

### Baseline characteristics

**Fig. 1:**
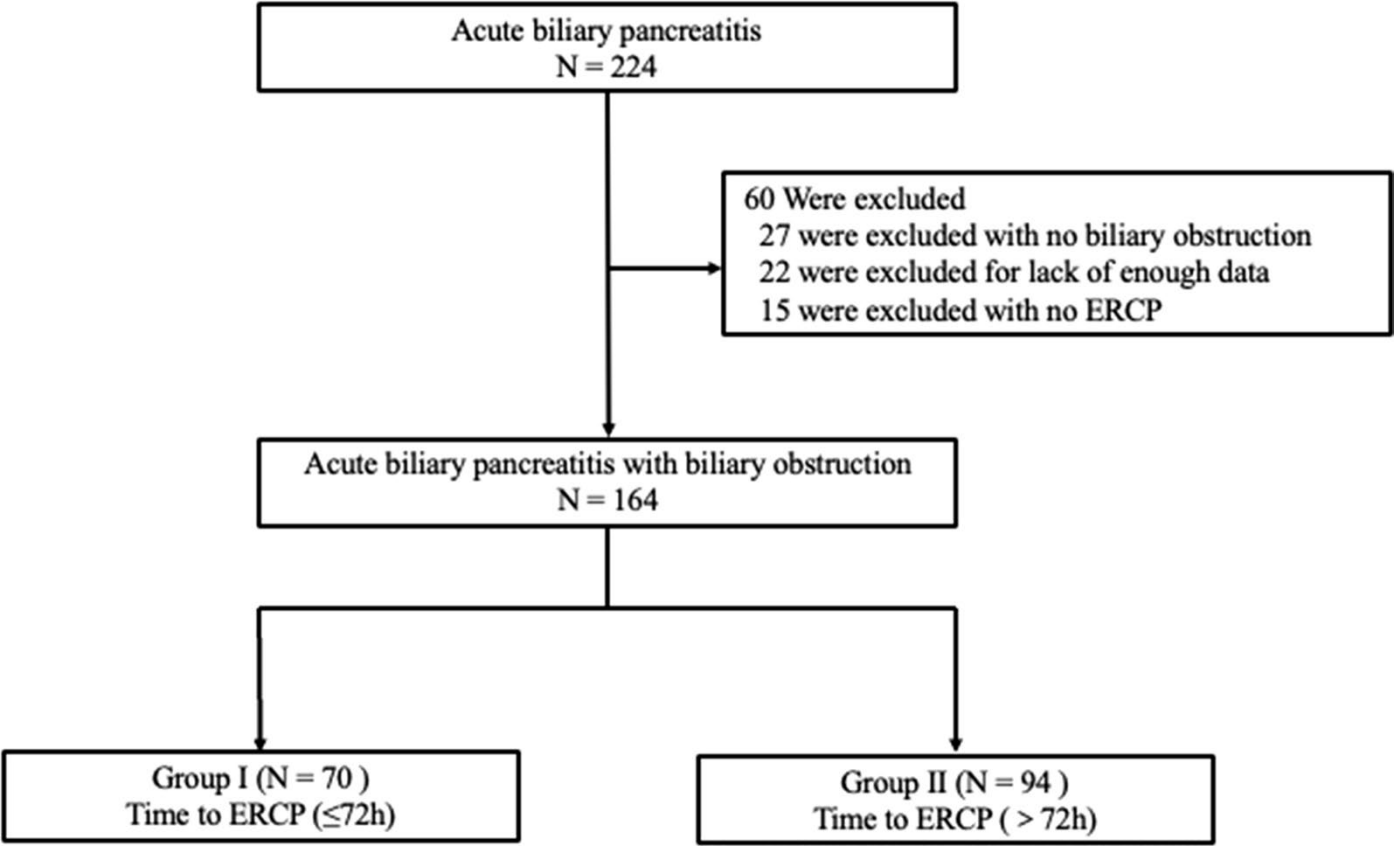
Flowchart detailing of the patients

In total, 164 patients were enrolled in our study (Fig. 1). The early ERCP group comprised 70 patients (39 men, 31 women), and the delayed ERCP group comprised 94 patients (42 men, 52 women). The patients’ mean age was 62.51 ± 14.38 years in the early ERCP group and 64.88 ± 16.00 years in the delayed ERCP group. The mean time from admission to ERCP was 44.07 ± 20.53 h in the early ERCP group and 163.83 ± 65.60 h in the delayed ERCP group. There was no significant difference in the amylase concentration, lipase concentration, or BISAP score between the two groups. The patients’ demographics and clinical characteristics are listed in Table 1. There were no significant differences in age, sex, ASA physical status, or laboratory data between the two groups.

**Table 1:** Characteristics of the patients at baseline

Characteristic	Group I (N = 70 )	Group II (N = 94)	*p*-value
Age (years)	62.51 ± 14.38	64.88 ± 16.00	0.33
Male/female, N (%)	39/31	42/52	0.21
Time of from admission to ERCP (hour)	44.07 ± 20.53	163.83 ± 65.60	0.00
Temperature (℃)	36.86 ± 0.59	37.01 ± 0.82	0.11
Total bilirubin (umol/L)	56.44 ± 48.02	41.63 ± 34.99	0.09
Direct bilirubin (umol/L)	39.14 ± 38.64	27.34 ± 28.84	0.09
BMI (kg/m^2^)	23.30 ± 2.17	23.38 ± 2.55	0.81
CRP (mg/L)	29.46 ± 44.33	24.38 ± 33.47	0.43
WBC (U/L)	11.01 ± 4.51	11.29 ± 4.18	0.18
PLT (U/L)	215.10 ± 71.90	199.60 ± 65.68	0.09
Amylase (U/L)	1534.13 ± 1502.45	1653.84 ± 1564.03	0.38
Creatine (U/L)	70.62 ± 21.21	70.00 ± 33.61	0.99
ALT (U/L)	220.11 ± 191.51	230.47 ± 219.35	0.73
AST U/L)	245.53 ± 217.38	236.31 ± 260.62	0.86
ALP (U/L)	176.17 ± 133.10	167.95 ± 136.14	0.74
γ-GT (U/L)	324.02 ± 246.87	391.3 ± 326.18	0.18
BISAP	1.02 ± 0.83	1.09 ± 0.86	0.72
Severity of AC			0.86
Mild	52	71	
Moderate	18	23	
Lipase (U/L)	605.03 ± 537.73	452.51 ± 395.14	0.26
Combines comorbid, N (%)			
Cardiovascular disease	28 (40)	35 (37.23)	0.72
Diabetes mellitus	10 (14.29)	11 (11.70)	0.62
COPD	3 (4.29)	3 (3.19)	0.71
MT	7 (10)	3 (3.19)	0.07
Others	1 (1.43)	3 (3.19	0.47
ASA(I/II/III/IV)	10/42/17/1	12/61/21/0	0.65
Previous abdominal surgery, N (%)	18(25.71)	14 (14.89)	0.08
Previous cholecystectomy, N (%)	9 (12.86)	5 (5.32)	0.09
Previous ERCP, N (%)	2 (2.86)	2 (2.13)	0.76

Continuous data were showed as mean ± SD

*ERCP* endoscopic retrograde cholangiopancreatography; *COPD* chronic obstructive pulmonary disease; *MT* malignant tumor; *BMI* body mass index; *ALP* alkaline phosphatase; *r-GT* γ-glutamyl transferase; *AST* aspartate aminotransferase; *ALT* alanine aminotransferase; *WBC* white blood cell; *CRP* C-reactive protein; *TB* total bilirubin; *DB* direct bilirubin; *BISAP* bedside index for severity in acute pancrieatitis

## Primary outcome and ERCP-related complications during waiting time

Primary outcome and ERCP-related measures were showed in Table 2. Technical success was achieved in 66 (94.29%) and 92 (97.87%) patients in the early and delayed ERCP groups, respectively (*p* = 0.43). Endoscopic sphincterotomy, endoscopic papillary balloon dilatation, and both were performed in 30, 25, and 11 patients, respectively, in the early ERCP group and in 36, 29, and 27 patients, respectively in the delayed ERCP group. There was no significant difference in the amylase or lipase concentration 24 h after ERCP between the two groups (*p* = 0.40 and *p* = 0.12, respectively). In the early ERCP group, four patients developed ERCP-related pancreatitis, one developed bleeding, and one developed cholangitis. In the delayed ERCP group, seven patients developed ERCP-related pancreatitis, one developed cholecystitis, and one developed cholangitis. There was no significant difference in these post-ERCP complications. No mortality occurred in either group. Laparoscopic cholecystectomy was performed after ERCP in 22 patients in the early ERCP group and 32 patients in the delayed ERCP group. There was no significant difference in the total hospital stay (12.76 ± 9.33 vs. 13.48 ± 4.53 days, *p* = 0.13). However, the length of hospital stay after ERCP was shorter in the delayed than early ERCP group (6.59 ± 3.37 vs. 10.94 ± 9.43 days, respectively; *p* = 0.00). There was no significant difference in patient cost between the two groups (24993.06 ± 9503.63 vs. 29547.13 ± 6815.92, *p* = 0.15).

**Table 2:** Primary outcome and ERCP-related measures

	Group I (n = 70)	Group II (n = 94)	*p*-value
Diameter of CBD (cm),	1.11 ± 0.95	1.13 ± 0.89	0.89
Cannulation success rate, N (%)	68(97.14)	92(97.87)	0.83
Technical success rate, N (%)	66 (94.29)	92(97.87)	0.43
Advanced cannulation techniques, N (%)	24(34.29)	16(17.02)	0.01
ERCP procedure, N (%)			0.18
EST	30 (42.86)	36 (38.30)	
EPBD	25 (35.71)	29 (30.85)	
EST + EPBD	11(15.71)	27 (28.72)	
Size of stone (mm)	10.25 ± 3.54	10.02 ± 2.71	0.49
Amylase(U/L)^a^	250.09 ± 408.12	200.20 ± 321.29	0.40
Lipase(U/L)^a^	166.25 ± 201.10	166.11 ± 199.52	0.12
Post-ERCP complications, N (%)	6 (8.57)	9 (9.57)	0.83
ERCP-related pancreatitis	4 (5.71)	7 (7.45)	0.90
Bleeding	1 (1.43)	0 (0)	0.43
Perforation	0 (0)	0 (0)	
Cholangitis	1 (1.43)	1 (1.06)	1.00
Cholecystitis	0 (0)	1 (1.06)	1.00
Mortality, N(%)	0 (0)	0 (0)	
LC after the ERCP, N(%)	22 (31.43)	32 (34.04)	0.72
Total length of hospital stays (day)^b^	12.76 ± 9.33	13.48 ± 4.53	0.13
Length of hospital stay after ERCP (day)^b^	10.94 ± 9.43	6.59 ± 3.37	0.00
Cost on the ERCP (¥)	12172.10 ± 4133.55	12435.38 ± 4178.54	0.33
Total hospital cost (¥) ^b^	24993.06 ± 9503.63	29547.13 ± 6815.92	0.15

Continuous data were showed as mean ± SD

*CBD* conmen bile duct; *EST* endoscopic sphincterotomy; *EPBD* endoscopic papillary balloon dilatation

^a^24 h post-ERCP

^b^The patients with LC were excluded

## Discussion

In this present study, we conducted a retrospective analysis to examine the clinical outcomes of different times to endoscopic intervention for ABP with non-severe cholangitis. Our study showed delayed ERCP performed > 72 h after admission has economic and safety outcomes similar to those of early ERCP for patients with concurrent ABP and non-severe cholangitis.

Performance of early ERCP is supported by the fact that the occurrence of biliary pancreatitis is often accompanied by the appearance of cholangitis. Early removal of the bile duct obstruction can reduce the reflux of pancreatic fluid and severity of pancreatitis. A guideline published in 2013 recommended the performance of ERCP within 24 h after admission for treatment of cholangitis in patients with ABP. However, the level of evidence was low. Additionally, some of the stones that caused ABP passed spontaneously into the duodenum, and most cases of ABP were self-limiting [12]. Moreover, early pancreatitis may cause duodenal edema, which may increase the difficulty of ERCP. Our study showed that delayed ERCP for ABP with non-severe AC had a technical success rate and safety outcome similar to those of early ERCP. However, early group has more difficulty cannulation than delayed. This may be related to factors such as duodenal papilla edema in the early stages of pancreatitis. Although there was no difference in the success rate of ERCP or incidence of postoperative complications between early and delayed ERCP, the early ERCP group did not gain an advantage in the length of hospital stay. Delayed ERCP can give patients more time to complete various examinations and, in some cases, can avoid unnecessary ERCP. In the early period of ABP, abdominal ultrasound has a low sensitivity for common bile duct [13]. Previous studies have confirmed the clinical value of Endoscopic ultrasound in the diagnosis of common bile duct stones [14]. Compared with abdominal ultrasound, EUS has higher specificity and sensitivity, and can detect some bile duct stones that cannot be detected by other tests. In this study, this part of the data is lacking because EUS is currently less performed in our hospital. Delayed ERCP did not increase the hospital stay or cost in this study. In clinical practice, the cost-effectiveness of ERCP for treatment of ABP must also be considered.

The time point adopted in our study was 72 h after admission. The time point varied among previous studies (admission or onset of symptoms). We believe that early acute pancreatitis is sometimes difficult to distinguish from acute cholangitis in the early stage. Many patients have difficulty accurately recalling the onset of their symptoms. Therefore, we believe that it is reasonable and feasible to adopt the time after admission.

Fölsch et al. [15] demonstrated that early ERCP is not superior to delayed ERCP for ABP without cholangitis. In the present study, acute cholangitis was defined according to the 2018 Tokyo guideline, which classifies acute cholangitis as mild, moderate, and severe [10]. According to the Tokyo guideline, urgent ERCP should be performed for severe cholangitis. For mild and moderate cholangitis, several studies have demonstrated that early ERCP and selective ERCP produced similar outcomes in terms of mortality and morbidity [16, 17]. In our study, most patients had non-severe cholangitis. Delayed ERCP did not increase the total hospital stay. Therefore, we believe that in the treatment of ABP with non-severe cholangitis, delayed ERCP does not increase the complication rate or length of hospital stay. Notably, there is a lack of clear diagnostic criteria for AC in ABP. We use the Tokyo guideline criteria, which have indicators regarding systemic inflammation that can be equally elevated in pancreatitis. This may pose some diagnostic difficulties. However, in combination with cholestasis and imaging evidence, it is still possible to make the diagnosis of acute cholangitis. Similarly, although previous studies have clearly defined post-ERCP complications, some of these descriptions remain ambiguous.

To the best of our knowledge, this study had the largest sample size to date, which is also one of the strengths of this research. However, this study also had two main limitations. First, it was a retrospective study, which may have led to selection bias. Additional high-quality studies are therefore required. Second, despite being the largest sample to date, our study sample was still relatively small.

In conclusion, this retrospective analysis showed that delayed ERCP performed > 72 h after admission has economic and safety outcomes similar to those of early ERCP for treatment of ABP with non-severe cholangitis.

## Data Availability

The datasets used and/or analyzed during the current study are available from the corresponding author on reasonable request.

## Abbreviations

ABP: Acute biliary pancreatitis
ERCP: Endoscopic retrograde cholangiopancreatography
BISA: Bedside index of severity in acute pancreatitis
